# True lumen stenting guided by intravascular ultrasound-confirmed wire repositioning from the subintimal space following balloon inflation during intravascular lithotripsy-induced coronary dissection: a case report

**DOI:** 10.1093/ehjcr/ytag433

**Published:** 2026-06-09

**Authors:** Hideo Tokuyama

**Affiliations:** Department of Cardiology, Kawaguchi Cardiovascular and Respiratory Hospital, 1-1-51 Maekawa, Kawaguchi City, Saitama 333-0842, Japan

**Keywords:** Intravascular lithotripsy, Complication, Coronary artery dissection, Subintimal wire migration, Intravascular ultrasound, Calcified lesion, Case report

## Abstract

**Background:**

Intravascular lithotripsy (IVL) is an effective modality for modifying severely calcified coronary lesions; however, IVL-induced coronary dissection may occasionally lead to inadvertent guidewire migration into the subintimal space. In such complex scenarios, real-time intravascular ultrasound (IVUS) is essential for identifying wire position, understanding dynamic wire–vessel interactions, and guiding safe stent deployment.

**Case summary:**

An 80-year-old woman with unstable angina underwent percutaneous coronary intervention for a severely calcified and tortuous left anterior descending artery. Intravascular lithotripsy was performed for calcium modification, but a longitudinal medial dissection flap developed, and the guidewire migrated into the subintimal space due to tortuosity-related wire bias. Multiple rewiring attempts using a double-lumen catheter and balloon support were unsuccessful. Real-time IVUS was used to evaluate the behaviour of a semi-compliant balloon positioned on the subintimal wire. Intravascular ultrasound demonstrated that balloon inflation redirected both the balloon and wire from the subintimal space into the true lumen, facilitated by anchoring of proximal and distal wire segments that remained within the true lumen. This confirmation enabled safe and accurate deployment of a drug-eluting stent in the true lumen.

**Discussion:**

Even when a guidewire migrates into the subintimal space during IVL-induced coronary dissection, balloon inflation may dynamically shift the device towards the true lumen due to anchoring by wire segments located proximally and distally within unaffected vessel segments. Real-time IVUS is indispensable for visualizing this dynamic behaviour and confirming whether balloon or stent expansion occurs within the true lumen. This IVUS-guided strategy may be valuable when conventional rewiring techniques fail.

Learning points

- Intravascular lithotripsy (IVL) can induce major coronary artery dissection in a few cases, occasionally resulting in wire migration into the subintimal space.
- Proximal–distal true-lumen wire support can shift wire bias during balloon inflation, redirecting a subintimal wire and balloon back into the true lumen.
- Real-time intravascular ultrasound (IVUS) is essential for confirming whether balloon expansion occurs in the true lumen or the subintimal space during this dynamic repositioning process.

## Introduction

Severely calcified coronary lesions continue to pose a major challenge in percutaneous coronary intervention (PCI).^1^ Adequate plaque modification is essential for achieving optimal stent expansion and long-term patency, yet the available tools each have distinct strengths and limitations. Rotational atherectomy (RA) and intravascular lithotripsy (IVL) are now widely regarded as complementary modalities: RA is generally preferred for balloon-uncrossable lesions, whereas IVL is suitable for balloon-crossable but undilatable calcification. Intravascular lithotripsy has gained increasing adoption because of its ease of use, predictable balloon behaviour, and favourable safety profile.^2^ However, despite its overall safety, IVL can occasionally induce coronary artery dissection. In rare cases, such dissections create an entry point that allows the guidewire to inadvertently migrate into the subintimal space. Stenting within this space carries substantial risks, including stent underexpansion, vessel perforation, and side branch occlusion.^3^

This report describes a unique scenario in which IVL-induced dissection, combined with pronounced vessel tortuosity and wire bias, resulted in guidewire migration into the subintimal space. We highlight how real-time intravascular ultrasound (IVUS) imaging was essential for confirming that balloon inflation redirected the device from the subintimal space into the true lumen, enabling safe stent deployment.

## Summary figure

**Figure:**
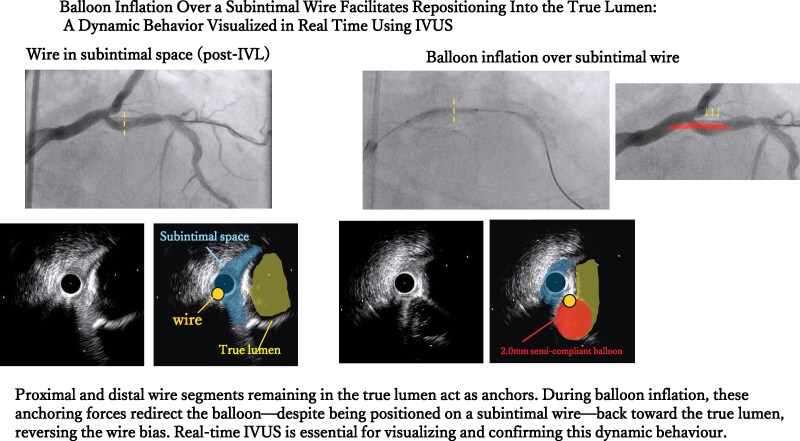


## Case presentation

An 80-year-old woman with diabetes mellitus, dyslipidaemia, hypertension, interstitial pneumonia, and autoimmune features presented with chest discomfort. Electrocardiography revealed ST-segment depression in leads V2–V6 and atrial fibrillation. High-sensitivity cardiac troponin T was elevated at 153 ng/L (reference < 14 ng/L). Suspecting unstable angina, elective coronary angiography was performed the following day.

**Figure 1:**
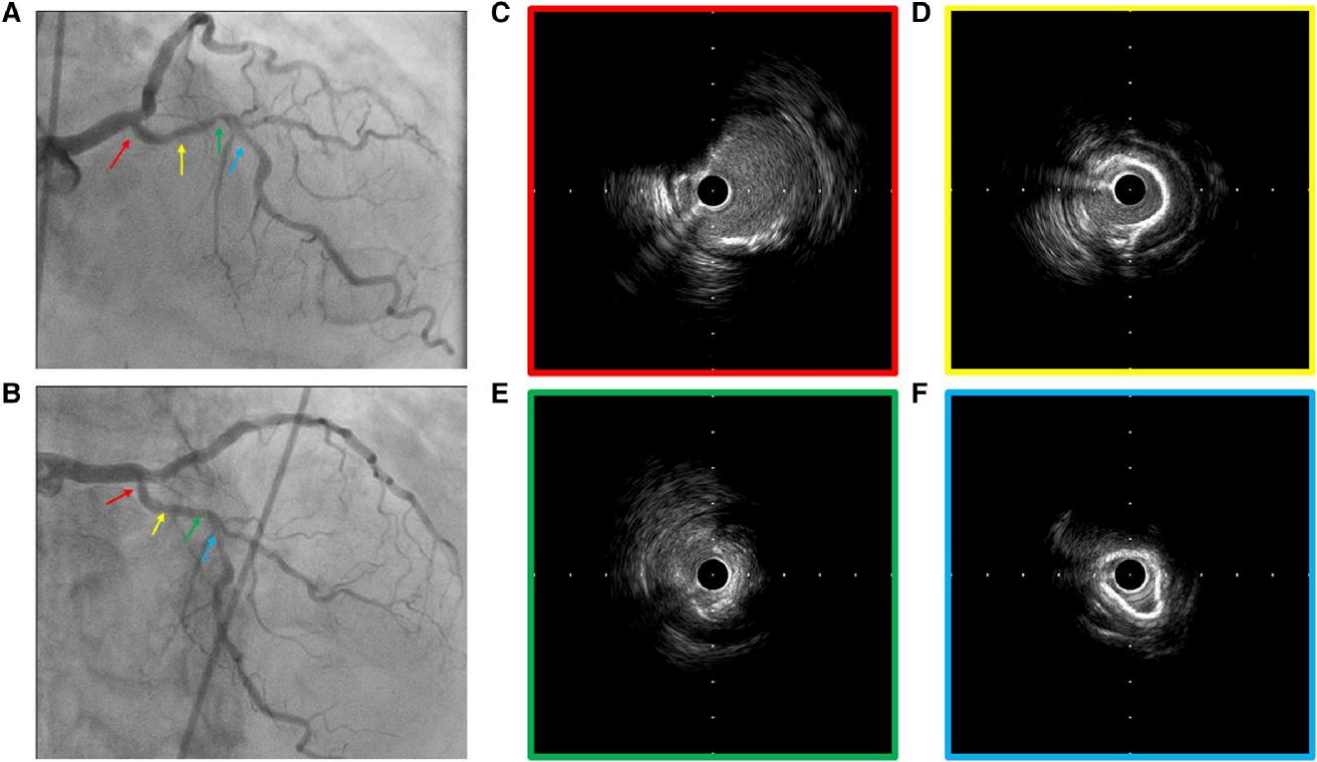
(*A* and *B*) Angiography images showing severe disease in the proximal to mid-left anterior descending artery, with marked tortuosity and heavy calcification. Arrows indicate anatomical locations within the proximal left anterior descending artery corresponding to intravascular ultrasound images. (*C*) Eccentric wire bias adjacent to calcified plaque (arrow). (*D*) Eccentric calcified lesion (arrow). (*E*) Soft plaque within a tight lesion (arrow). (*F*) Concentric calcified lesion (arrow).

Angiography revealed severe, tortuous, heavily calcified stenosis in the proximal–mid-LAD (*Figure 1*). Percutaneous coronary intervention was initiated using a 6 Fr guiding catheter. Intravascular ultrasound demonstrated eccentric proximal and concentric mid-LAD calcification. Because the lesion was balloon crossable but resistant to dilation, IVL was selected for calcium modification. To protect against potential major dissection, an additional floppy wire was advanced into a large diagonal branch. Intravascular ultrasound measurements showed a distal reference external elastic membrane (EEM) diameter of 3.5 mm. A 3.0 × 12 mm IVL balloon was inflated at 400 kPa, delivering eight cycles of 80 pulses to the mid- and proximal LAD.

**Figure 2:**
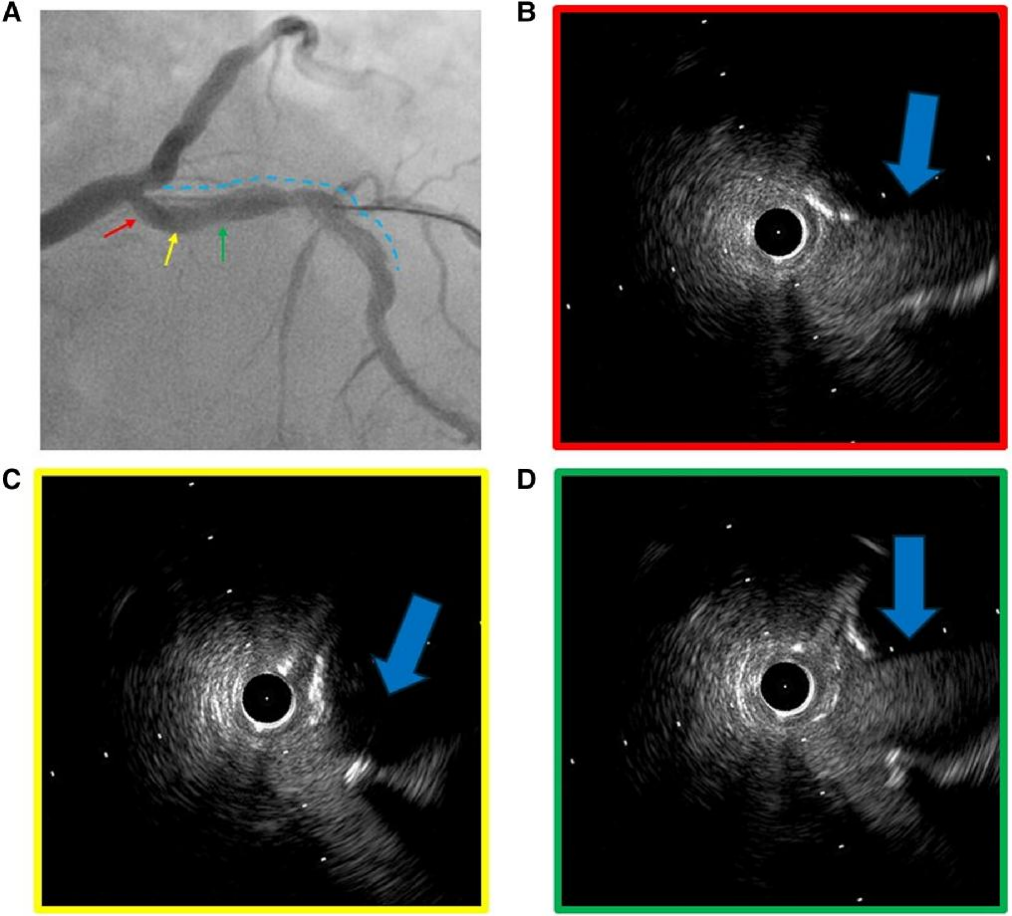
(*A*) Angiographic image following intravascular lithotripsy performed in the proximal to mid-left anterior descending artery (dashed line), showing a Type C coronary artery dissection in the proximal left anterior descending segment. Arrows indicate anatomical locations corresponding to intravascular ultrasound images. Arrows also denote the true lumen. (*B*) Intravascular ultrasound image showing medial disruption and migration of both the wire and intravascular ultrasound catheter into the subintimal space. (*C*) Intravascular ultrasound image demonstrating the wire and catheter located behind the calcified plaque, within the subintimal space. (*D*) Intravascular ultrasound image confirming the wire’s position in the subintimal space adjacent to the true lumen.

Although the balloon expanded appropriately, subsequent angiography revealed a Type C dissection in the proximal LAD with guidewire migration from the true lumen into the subintimal space. Intravascular ultrasound confirmed medial disruption and demonstrated that both the guidewire and IVUS catheter had entered the subintimal space external to the calcified plaque. Rather than a single focal entry point, IVL created a longitudinal medial dissection flap extending along the vessel’s long axis, resulting in a continuous subintimal plane. The wire did not enter through an isolated defect; instead, it migrated into the subintimal lumen by tracking along this elongated flap, which provided a permissive pathway. The pronounced tortuosity of the proximal LAD further imposed a strong directional wire bias, and once the longitudinal subintimal plane was formed, its orientation aligned with this bias. Consequently, the wire repeatedly followed the length of the subintimal space rather than remaining within the true lumen (*Figure 2*).

Because stenting within the subintimal space carries significant risks, true-lumen rewiring was attempted using an additional floppy wire. The first attempt was performed with a double-lumen catheter. A second attempt was made during small-balloon inflation at the proximal dissection site to occupy the subintimal space and redirect the wire towards the true lumen. Although both attempts initially appeared successful, the wire consistently migrated back into the subintimal space when the double-lumen catheter was withdrawn or the balloon was deflated. This difficulty was attributed to the alignment of the dissection flap with the vessel’s tortuous anatomy, which reinforced the wire bias towards the subintimal channel.

**Figure 3:**
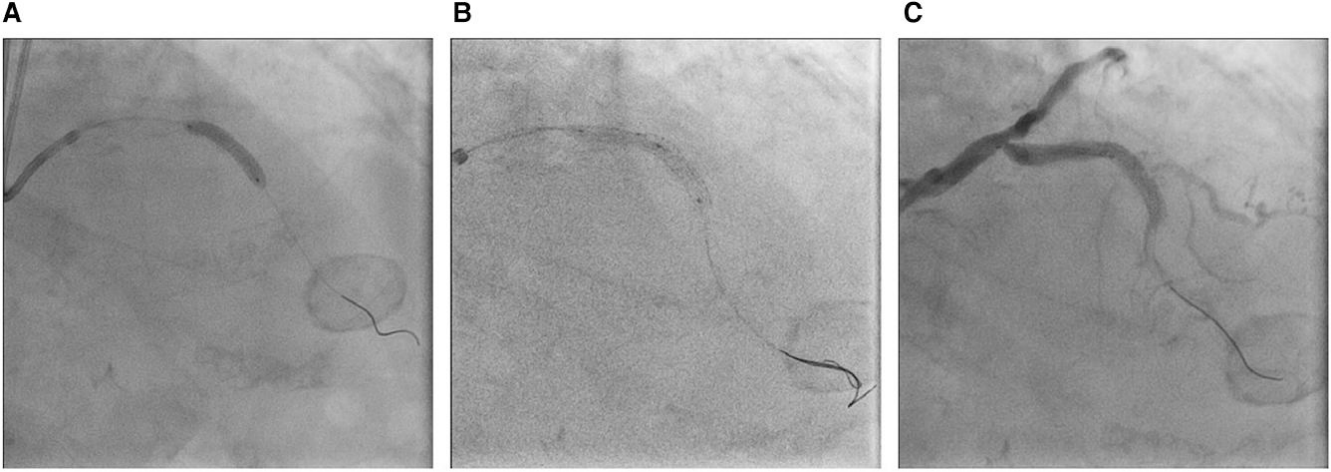
(*A*) A 3.5 × 16 mm drug-eluting stent implanted in the mid-left anterior descending artery. (*B*) Real-time intravascular ultrasound evaluation of the behaviour and dynamics of a semi-compliant 2.0 mm balloon. (*C*) A 4.0 × 18 mm drug-eluting stent intentionally deployed in the true lumen over a wire that had migrated into the subintimal space.

During these rewiring attempts, the proximal dissection propagated distally, creating a new flap in the mid-LAD and compromising the lumen. Because the wire in the mid-LAD fortunately remained within the true lumen, a 3.5 × 16 mm drug-eluting stent (DES) was first implanted in the distal portion of the dissected segment to stabilize the vessel and prevent further medial dissection propagation. Intravascular ultrasound confirmed that this stent was deployed within the true lumen (*Figure 3A*).

After stabilizing the mid-LAD, attention returned to the proximal LAD, where true-lumen stenting was still required. At this stage, real-time IVUS was used to evaluate whether balloon inflation could redirect the device from the subintimal space into the true lumen. A 2.0 × 15 mm semi-compliant balloon was mounted on one wire, while the IVUS catheter was advanced over the other. Because simultaneous insertion of both devices was not feasible with a 6 Fr system, all intracoronary devices were removed, and both the 6 Fr sheath and guiding catheter were exchanged for an 8 Fr system over a standard 0.035-inch wire. This provided sufficient guiding-catheter lumen to advance the IVUS catheter and balloon simultaneously.

**Figure 4:**
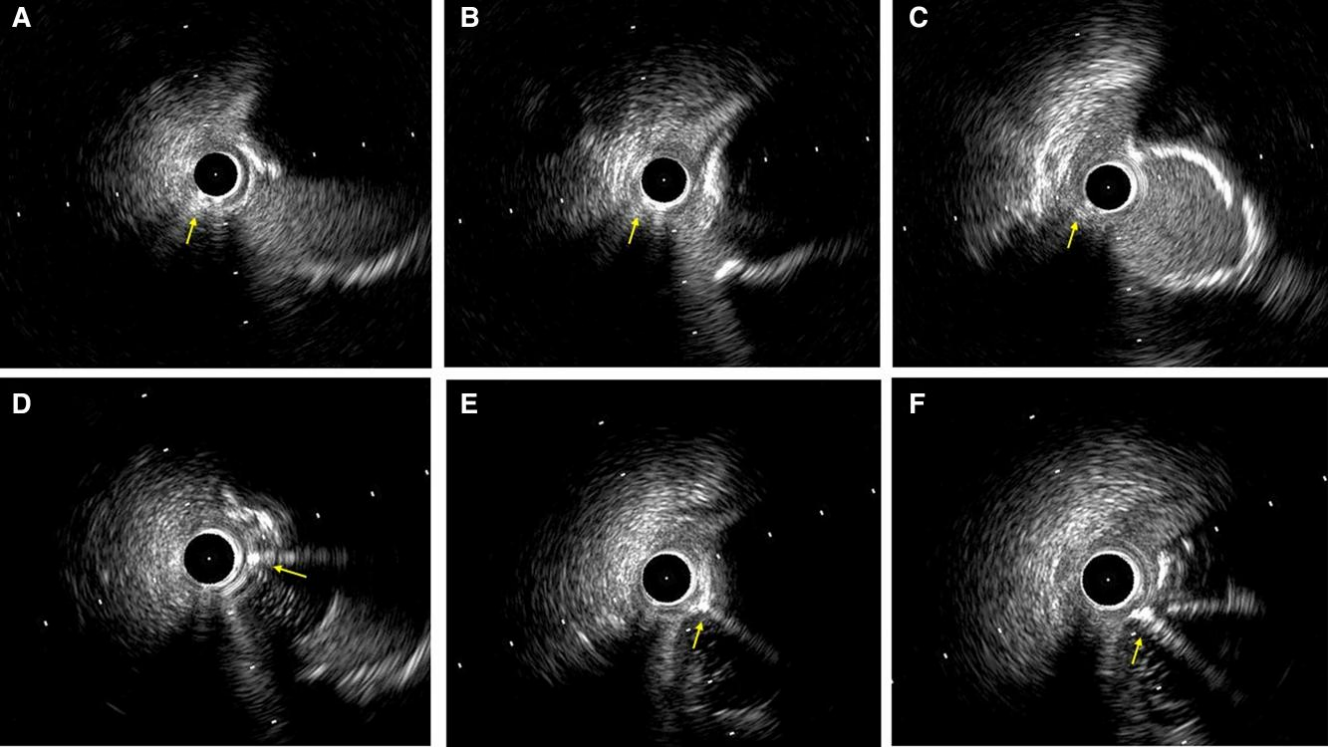
(*A–C*) Intravascular ultrasound images obtained sequentially from proximal to distal segments within the proximal left anterior descending, showing migration of both the intravascular ultrasound catheter and an additional wire into the subintimal space. (*D–F*) Corresponding intravascular ultrasound images following balloon inflation demonstrating repositioning of a semi-compliant 2.0 mm balloon and an additional wire from the subintimal space into the true lumen. The arrows indicate the additional wire.

**Figure 5:**
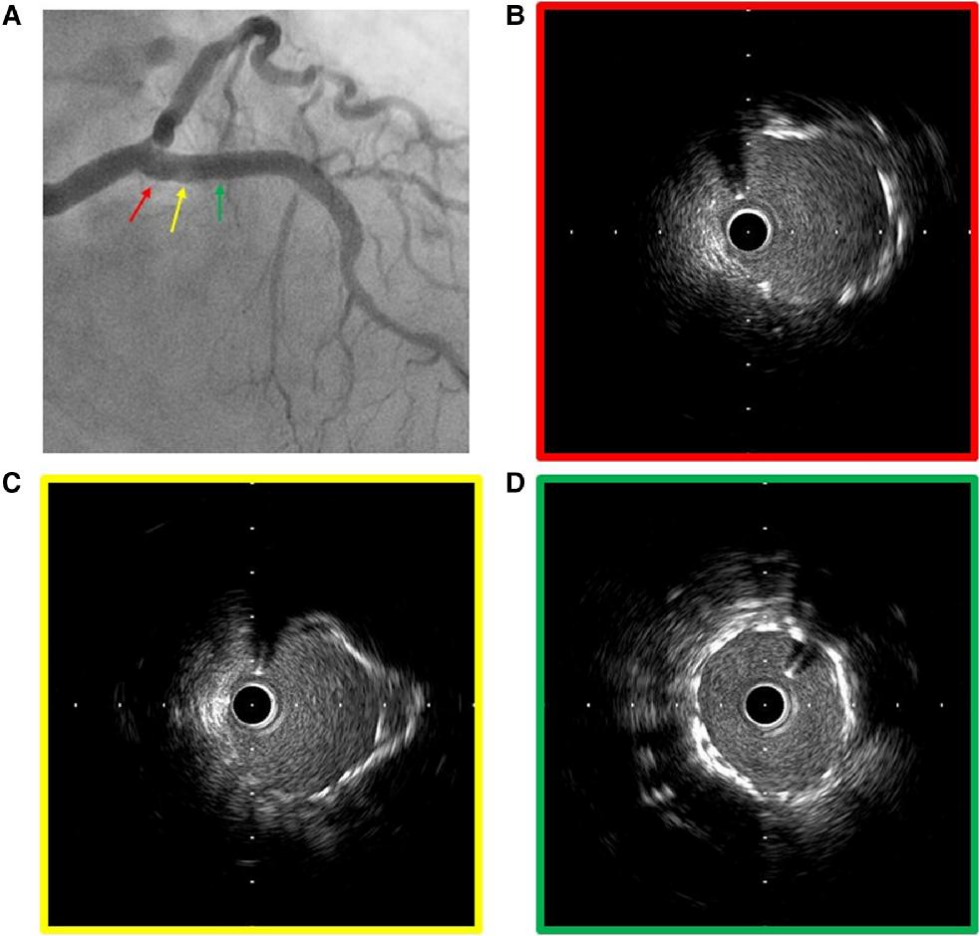
(*A*) Final coronary angiography following stent implantation. (*B–D*) Intravascular ultrasound images obtained sequentially from the proximal to distal segments within the proximal left anterior descending, confirming drug-eluting stent deployment in the true lumen at the site where the wire had migrated into the subintimal space. Post-dilatation resulted in good symmetric stent expansion and acceptable lumen gain.

Intravascular ultrasound confirmed that the balloon-mounted wire was initially located in the subintimal space (see Supplementary material online, *Video S1*). However, during balloon inflation, both the wire and balloon shifted into the true lumen (*Figures 3B* and  *4*; Supplementary material online, *Video S2*). Based on this real-time confirmation, a DES was delivered over the wire that had migrated from the subintimal space and was successfully deployed within the true lumen (*Figure 3C*; Supplementary material online, *Video S3*). Additional stenting from the left main trunk to the proximal LAD was performed to treat residual dissection, achieving satisfactory lumen gain and symmetric stent expansion (*Figure 5*).

## Discussion

This case illustrates how IVL-induced dissection, combined with tortuosity-related wire bias, can lead to persistent subintimal wire migration. The mechanism is best understood as an interaction between the dissection flap created by IVL and the pre-existing directional bias imposed by the vessel’s tortuosity. Once the longitudinal dissection flap aligned with this bias, the wire preferentially tracked into the subintimal space, making true-lumen recrossing difficult despite multiple conventional techniques.

A key observation in this case is that even when IVUS confirms that the wire lies in the subintimal space, balloon expansion may still occur in the true lumen. This counterintuitive behaviour is explained by the anchoring effect of wire segments that remain within the true lumen proximally and distally. These segments create a stabilizing vector that redirects the balloon or stent towards the true lumen during inflation. Angiography alone cannot reliably distinguish these interactions, as it does not depict the spatial relationship between the wire, dissection plane, and true lumen.

Real-time IVUS was therefore essential for procedural decision-making. It allowed direct visualization of the dynamic interaction between the wire, balloon, and vessel wall, confirming that balloon expansion occurred within the true lumen before stent deployment. This strategy may be particularly valuable in complex dissections where wire migration into the subintimal space occurs and conventional rewiring techniques fail. The supplementary IVUS videos further demonstrate the dynamic behaviour that informed these decisions and underscore the importance of real-time imaging in complex PCI.

## Conclusion

This case demonstrates that an inflated balloon positioned over a wire located in the subintimal space may dynamically shift into the true lumen during balloon inflation, particularly when the device is anchored by wire segments that remain within the true lumen proximally and distally. Real-time IVUS was essential for confirming this behaviour and ensuring safe stent deployment. This IVUS-guided strategy may serve as a practical and effective option in complex coronary dissections when conventional rewiring techniques fail.

## Supplementary Material

ytag433_Supplementary_Data

## Data Availability

The data underlying this article will be shared on reasonable request to the corresponding author.

## Lead author biography

Hideo Tokuyama graduated from Nippon Medical School in 2009 and completed his postgraduate training at Nippon Medical School Hospital. Since 2015, he has been working at Kawaguchi Cardiovascular and Respiratory Hospital. His clinical expertise includes ischaemic heart disease and structural heart disease.

## Notes

**Consent:** Written informed consent was obtained from the patient for publication of this case report and all accompanying images, in line with the COPE guidelines.
